# Supplementary material for: Social isolation shortens lifespan through oxidative stress in ants
Source: Nat Commun. 2023 Sep 27;14:5493. doi: 10.1038/s41467-023-41140-w (PMC10533837; doi:10.1038/s41467-023-41140-w)
Supplement: Supplementary file 1 — Supplementary Information [file 41467_2023_41140_MOESM1_ESM.pdf]

Supplementary Information

Social isolation shortens lifespan through oxidative stress in ants

Akiko Koto[1,2*], Makoto Tamura[3], Pui Shan Wong[2], Sachiyo Aburatani[1,2], Eyal Privman[4], Céline Stoffel[5], Alessandro Crespi[6], Sean Keane McKenzie[5], Christine La Mendola[5], Tomas Kay[5], Laurent Keller[5†*]

[1]Bioproduction Research Institute, National Institute of Advanced Industrial Science and Technology, Tsukuba, 305-8566, Ibaraki, Japan

[2]Computational Bio Big Data Open Innovation Laboratory (CBBD-OIL), National Institute of Advanced Industrial Science and Technology, Tsukuba, 305-8566, Ibaraki, Japan

[3]NeuroDiscovery Lab, Mitsubishi Tanabe Pharma America, Cambridge, MA 02139, USA

[4]University of Haifa, Institute of Evolution, Department of Evolutionary and Environmental Biology, Haifa, 3498838, Israel

[5]University of Lausanne, Department of Ecology and Evolution, Lausanne, CH-1015, Switzerland

[6]Biorobotics Laboratory, Ecole Polytechnique Fédérale de Lausanne, Lausanne, CH-1015, Switzerland

[†] Present address: Social Evolution Unit, Cornuit 8, BP 855, Chesières, CH-1885, Switzerland

*Address correspondence to Akiko Koto, a-koto@aist.go.jp and Laurent Keller, Laurent.keller01@gmail.com

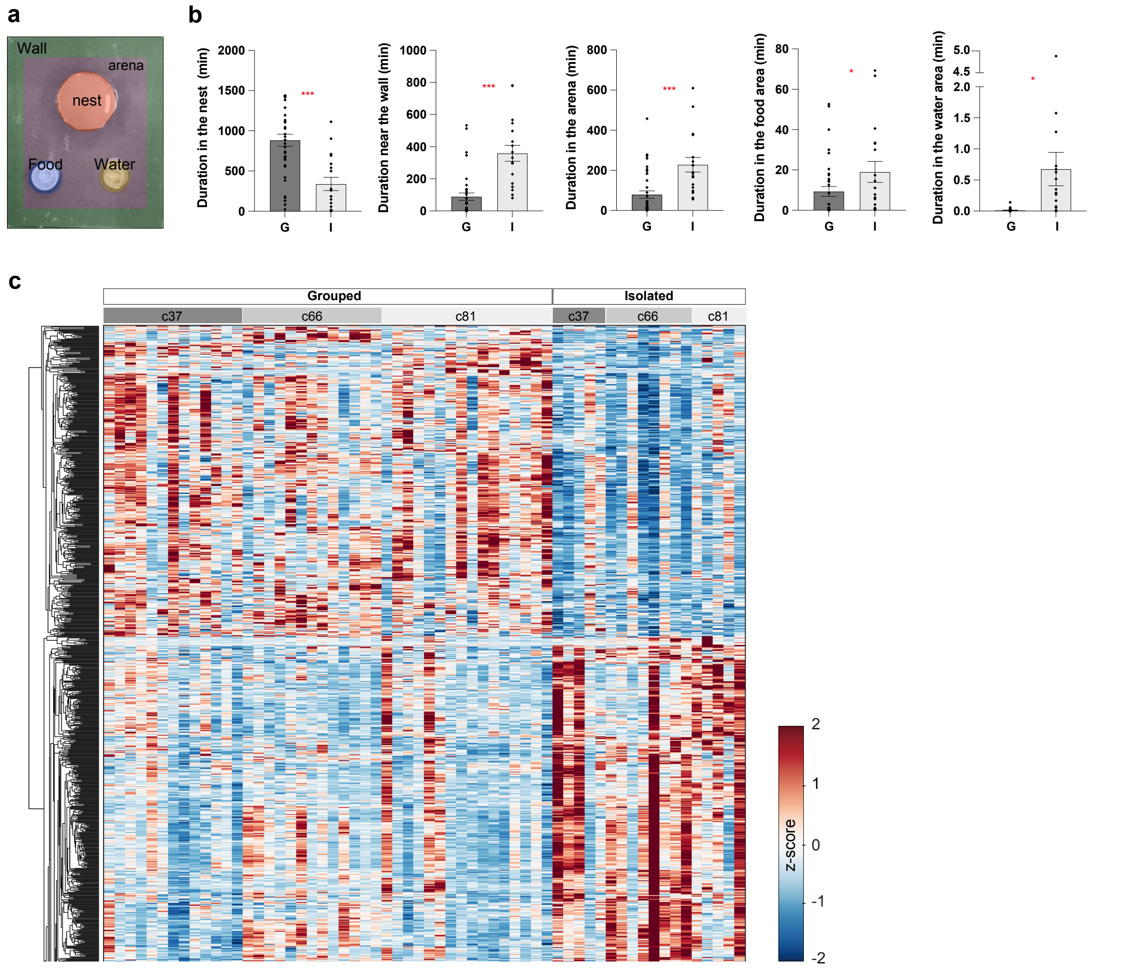

Supplementary Figure 1. Behavioral setting and heatmap with hierarchical clustering of the 894 genes differentially expressed by social treatment

a Definition of the five regions calculated, including nest (red), wall (green), arena, food region (blue) and water (yellow) region. **b** Time (min/day, mean ± SEM with all data points) spent in the nest (p<0.0001), near the wall (p<0.0001), in the arena (p<0.001), food area (p=0.049), and water area (p=0.013) for grouped (dark gray, n=36) and isolated workers (light gray, n=18). The effect of treatment on behavior was tested using generalized linear mixed models (GLMMs) and subsequent ANOVA tests: *p<0.05; ***p<0.001. **c** Each row corresponds to one gene and each column corresponds to one sample. Colony ID is indicated at the top of each column.

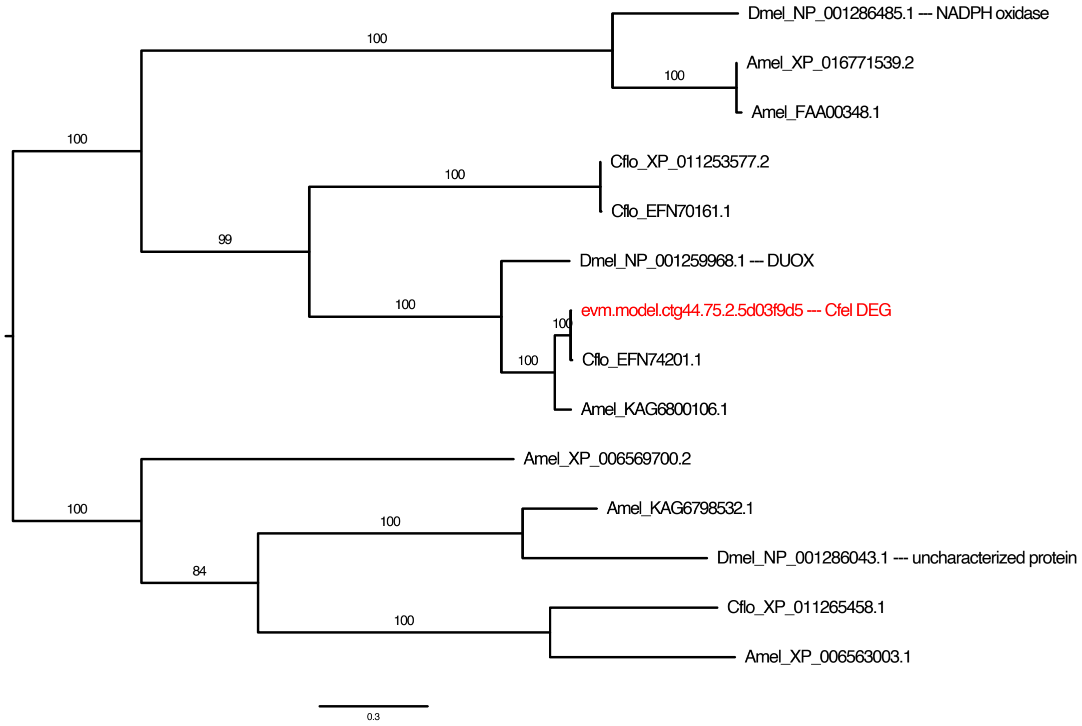

Supplementary Figure 2. Phylogeny of *DUOX* gene

Phylogeny of *DUOX* in *Camponotus fellah* (*Cfel* DEG in red) and its homologs (orthologs and closest paralogs) in *Camponotus floridanus* (*Cflo*), *Drosophila melanogaster* (*Dmel*), and *Apis mellifera* (*Amel*).

a

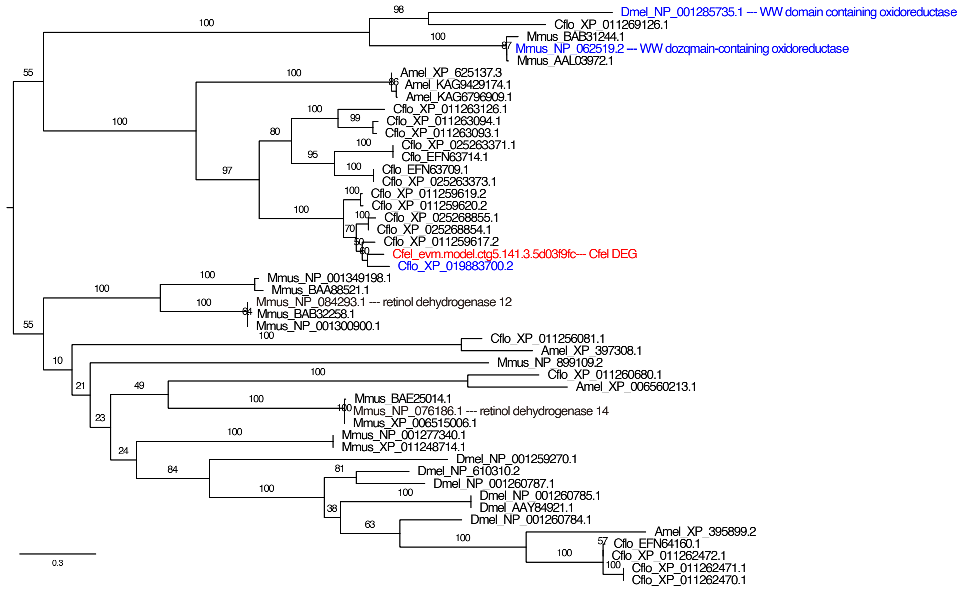

b

```
Cfel_evm.model.ctg5.141.3.5d03      ----------------------------------------------------------------------------------------------------
Cflo_XP_019883700.2                 ----------------------------------------------------------------------------------------------------
Dmel_NP_001285735.1                 -----MIALPDTDSEDELPPGWEERATDDGTVCYVNQQGKTSQWTHPRTGRSKRITGELPLGWEKYYDEQGKRFMFLNKETQQRTNVDPRLAFAVEEPTQ
Mmus_NP_062519.2                    MAALRYAGLDDTDSEDELPPGWEERTTKDGWVYYANHTEEKTQWEHPKTGKRKRVAGDLPYGWEQETDENGQ-VFFVDHINKRTTYLDPRLAFTVDD-NP
                                                   WW Domain

Cfel_evm.model.ctg5.141.3.5d03      -------------------------GKTVVITGASSGIGKETARDFYGRGARVILACINMEEANEAVKDIKNNPSSRIKKDDYQNGAGELAIYFLNLCSL
Cflo_XP_019883700.2                 ----------MWFLKKLCTSNARLEGKTVVITGASSGIGKETARDFYGRGARVILACINMEEANEAVKDIKSNPPFRIKKDEYQNGAGELAIYFLNLCSL
Dmel_NP_001285735.1                 NVAQVRQRFDSCSTALQVLHGKDLHGRTALITGANCGIGYETARSLAHHGCEIIFACRNRSSAEAAIERIAQ---------ERPAARSRCRFAALDLSSL
Mmus_NP_062519.2                    TKPTTRQRYDGSTTAMEILQGRDFTGKVVLVTGANSGIGFETAKSFALHGAHVILACRNLSRASEAVSRILE---------EWHKAK--VEAMTLDLAVL
                                                             *:..::***..*** ***:.:  :*..:*:** * . *. *:. * .         :   .        *:*. *

Cfel_evm.model.ctg5.141.3.5d03      KSVKDCAKNLLMKEAAIHILVNNAGVAAIPYEKTEDGIETTFQTNHLGHFLLTLLLLPKMQSSSPGCRIVNVSSIGHIFGDIDFDDINLEKS--------
Cflo_XP_019883700.2                 KSVKDCAKNLLMKEAAIHILVNNAGVVAISYEKTEDGIETTFQTNHLGHFLLTLLLLPKMQSSSPGCRIVNISSIGHIFGDIDFDDINLEKS--------
Dmel_NP_001285735.1                 RSVQRFVEEIKQSVSHIDYLILNAGVFALPYTRTVDGLETTFQVSHLSHFYLTLQLETLFDYKT---RIIVLSSESHRFANLPVEN--LAVHHLSPPPEK
Mmus_NP_062519.2                    RSVQHFAEAFKAKNVSLHVLVCNAGTFALPWGLTKDGLETTFQVNHLGHFYLVQLLQDVLCRSSP-ARVIVVSSESHRFTDINDSSGKLDLSRLSPPRSD
                                    :**:  .: :  .   :. *: ***. *:.:  * **:*****..**.** *.  *   :  .:   *:: :** .* * ::  ..  *

Cfel_evm.model.ctg5.141.3.5d03      YGPLKSYFQSKLANILFTRELARRLNKANIHGINVYSLHPGNIPTDITRHAFSTFFPGASYFYNFLSWVSPWVLPWLKTVEEGAQTTIYCSVDEKTANET
Cflo_XP_019883700.2                 YRPLKSYFQSKLANILFTRELARRLNKANVHGINVYSLHPGVMPTKVTRHASSTIFPGGSYAYKFVLWVLPWAF---KTVEEGAQTTIYCSVDEKTANDT
Dmel_NP_001285735.1                 YWSMMAYNNAKLCNVLFAQELAQRWKQR---GISVFSLHPGNMVSSDLSRN-----------YWFYRLLFAIVRPFTKSLQQAAATSIYCATANELTGLS
Mmus_NP_062519.2                    YWAMLAYNRSKLCNILFSNELHRRLSPR---GVTSNAVHPGNMMYSAIHRN-----------SWVYKLLFTLARPFTKSMQQGAATTVYCAVAPELEGLG
                                    * .: :* .:**.*:**:.** :* .     *:.  ::*** :  .   :              .   : . .    *::::.* *::**:.  :  .

Cfel_evm.model.ctg5.141.3.5d03      GLYYS-----------------------------------------------
Cflo_XP_019883700.2                 GLYYSDCNIVNPRRKVTNDEYAKNLWDVSCKLLNLEPEENFTKFLQTVSRQI
Dmel_NP_001285735.1                 GLYFNNCFFCEPSKLSKSAALQQQLWKLSENLIAELVEQEQH----------
Mmus_NP_062519.2                    GMYFNNCCRCLPSEEAQSEETARALWELSERLIQDRLGSPSS----------
                                    *:*:.
```

Supplementary Figure 3. Phylogeny of *Wwox-like 1* gene

a Phylogeny of *Wwox-like 1* in *Camponotus fellah* (*Cfel* DEG in red) and its homologs (orthologs and closest paralogs) in *Camponotus floridanus* (*Cflo*), *Drosophila melanogaster* (*Dmel*), *Apis mellifera* (*Amel*), and *Mus musculus* (*Mmus*). **b** Amino acid sequence alignment of *Wwox-like 1* in *Cfel* (in red in **a**) and *Wwox* from *Dmel* and *Mmus*, and *Wwox-like 1* in *Cflo* (in blue in **a**). Position of Wwox domain (red) is marked below the alignment blocks.

a

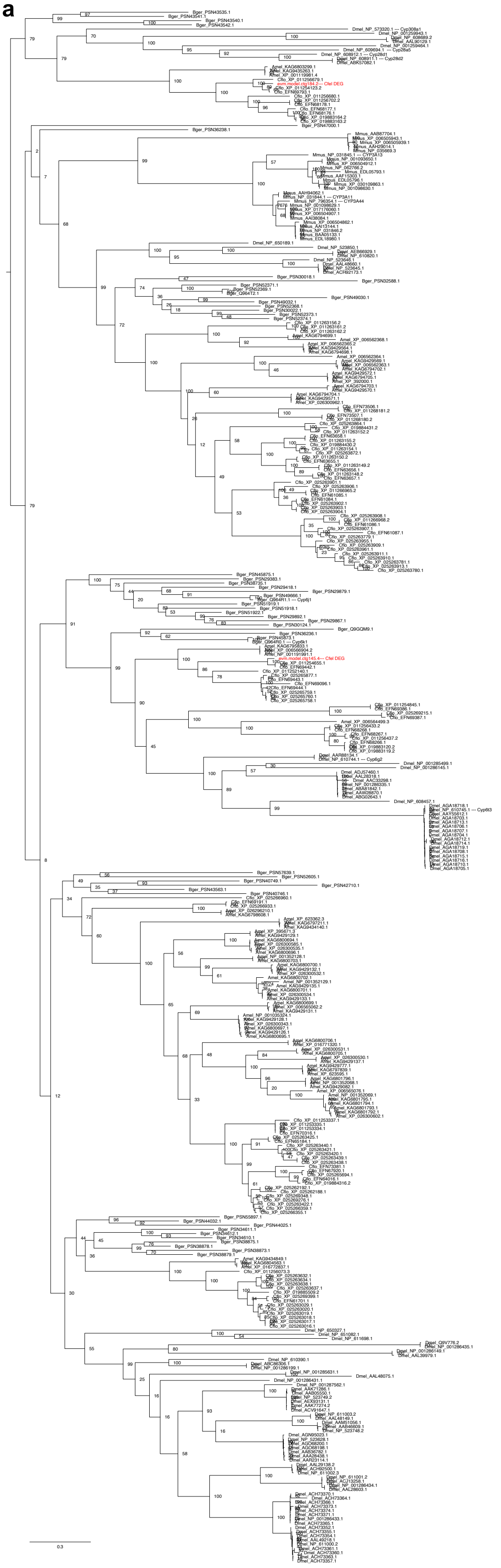

b

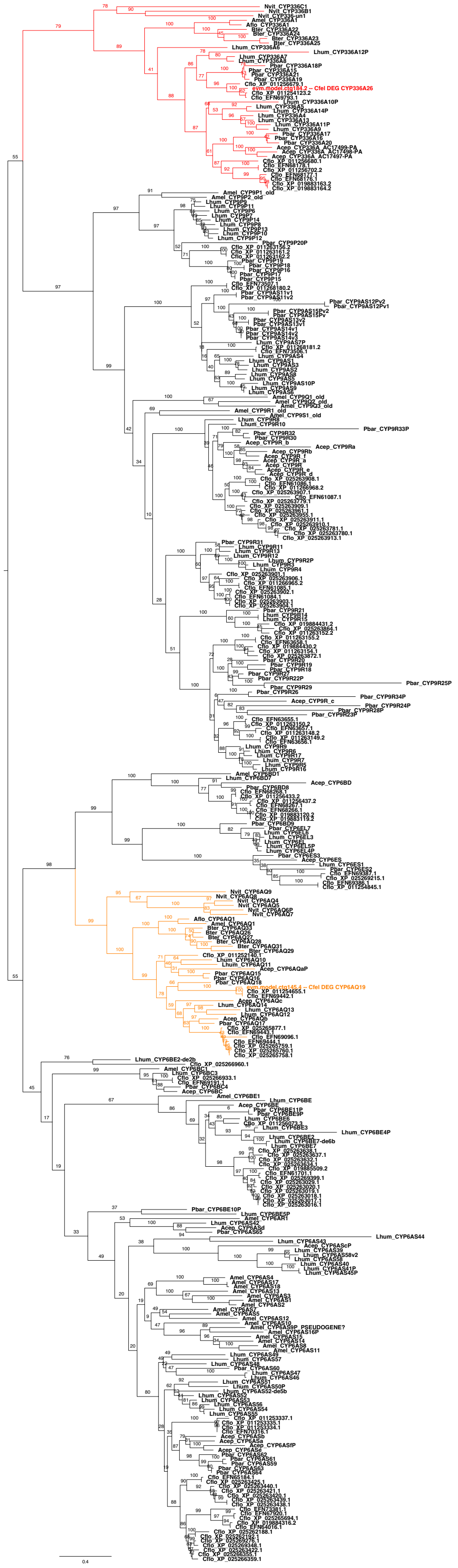

Supplementary Figure 4. Phylogeny of *CYP* genes

a Phylogeny of the two *CYP* DEGs in *Camponotus fellah* (*Cfel* DEG in red) and their homologs (orthologs and closest paralogs) in *Camponotus floridanus* (*Cflo*)*, Drosophila melanogaster* (*Dmel*)*, Apis mellifera* (*Amel*)*, Blattella germanica* (*Bger*)*,* and *Mus musculus* (*Mmus*). **b** Phylogeny of the two *CYP* DEGs in *Cfel* (red) and CYPs in clan 3 according to Nelson's cytochrome P450 website[1] from *Amel*, *Cflo*, *Nasonia vitripennis (Nvit), Bombus terrestris (Bter), Atta cephalotes* (*Acep*), *Linepithema humile* (*Lhum*), and *Pogonomyrmex barbatus* (*Pbar*). Relevant clades are highlight by colored branches (*CYP336A26* in red, and *CYP6AQ19* in orange).

Supplementary Table 1. Lists of primers used for qRTPCR analysis

Gene name	Primer Type	Sequence (5' to 3')
ef1a	Forward	CCACCAGGCCGACTGATAAG
	Reverse	AGGTACTGTTCCAATACCACCAATC
rp2	Forward	TGGGTCATCGCGTCAAAGT
	Reverse	GCGATGTGCAGCTCAGGTT
CYP336A26	Forward	TCCTCGATATTGGGAGCATC
	Reverse	TGTGTAGCCACCATTTCCAA
DUOX	Forward	CGGCATTGCTAAATGAGGAT
	Reverse	GATTCGAGTCCCACCAGGTA
Wwox-like 1	Forward	TTCCCCAGGTTGCAGAATAG
	Reverse	ACGAGCGAGTTCCCTTGTAA
CYP6AQ19	Forward	ACAACGTGTCACCACCAAGA
	Reverse	AGCTCCAAAAATGCCATACG

Supplementary Reference

1 Nelson, D. R. Cytochrome P450 Homepage (The University of Tennessee Health Science Center). https://drnelson.uthsc.edu/
