# Supplementary material for: Social isolation shortens lifespan through oxidative stress in ants
Source: Nat Commun. 2023 Sep 27;14:5493. doi: 10.1038/s41467-023-41140-w (PMC10533837; doi:10.1038/s41467-023-41140-w)
Supplement: Supplementary file 3 — Description of Additional Supplementary Files [file 41467_2023_41140_MOESM3_ESM.pdf]

Description of Additional Supplementary Files

File Name: Supplementary Data 1

Description: DEGs with oxidoreductase activity in module 1. The results of blast annotation against *Dmel*, $\log_2$ FC (grouped/isolated) and q-value with the false discovery rate correction are listed. DEGs up-regulated in isolated ants are shown on the top, and DEGs down-regulated in isolated ants are shown on the bottom. DEGs tested in Fig. 2 are highlighted in red.

File Name: Supplementary Data 2

Description: Summary of Data Availability deposited in the repositories.

File Name: Supplementary Code 1

Description: Statistic analysis from Fig. 1 to 4 and Supplementary Fig. 1.

File Name: Supplementary Code 2

Description: faa data for Supplementary Fig. 2

File Name: Supplementary Code 3

Description: raxml file for Supplementary Fig. 2

File Name: Supplementary Code 4

Description: faa data for Supplementary Fig. 3

File Name: Supplementary Code 5

Description: raxml file for Supplementary Fig. 3

File Name: Supplementary Code 6

Description: faa data for Supplementary Fig. 4a

File Name: Supplementary Code 7

Description: raxml file for Supplementary Fig. 4a

File Name: Supplementary Code 8

Description: faa data for Supplementary Fig. 4b

File Name: Supplementary Code 9
Description: raxml file for Supplementary Fig. 4b
