# Supplementary material for: Social isolation shortens lifespan through oxidative stress in ants
Source: Nat Commun. 2023 Sep 27;14:5493. doi: 10.1038/s41467-023-41140-w (PMC10533837; doi:10.1038/s41467-023-41140-w)
Supplement: Supplementary file 15 — Reporting Summary [file 41467_2023_41140_MOESM15_ESM.pdf]

Corresponding author(s): Akiko Koto
Last updated by author(s): Apr 4, 2023

Reporting Summary

Nature Portfolio wishes to improve the reproducibility of the work that we publish. This form provides structure for consistency and transparency in reporting. For further information on Nature Portfolio policies, see our Editorial Policies and the Editorial Policy Checklist.

Statistics

For all statistical analyses, confirm that the following items are present in the figure legend, table legend, main text, or Methods section.

n/a	Confirmed	
☐	☒	The exact sample size (*n*) for each experimental group/condition, given as a discrete number and unit of measurement
☐	☒	A statement on whether measurements were taken from distinct samples or whether the same sample was measured repeatedly
☐	☒	The statistical test(s) used AND whether they are one- or two-sided<br>*Only common tests should be described solely by name; describe more complex techniques in the Methods section.*
☐	☒	A description of all covariates tested
☐	☒	A description of any assumptions or corrections, such as tests of normality and adjustment for multiple comparisons
☐	☒	A full description of the statistical parameters including central tendency (e.g. means) or other basic estimates (e.g. regression coefficient) AND variation (e.g. standard deviation) or associated estimates of uncertainty (e.g. confidence intervals)
☐	☒	For null hypothesis testing, the test statistic (e.g. *F*, *t*, *r*) with confidence intervals, effect sizes, degrees of freedom and *P* value noted<br>*Give P values as exact values whenever suitable.*
☒	☐	For Bayesian analysis, information on the choice of priors and Markov chain Monte Carlo settings
☒	☐	For hierarchical and complex designs, identification of the appropriate level for tests and full reporting of outcomes
☐	☒	Estimates of effect sizes (e.g. Cohen's *d*, Pearson's *r*), indicating how they were calculated

Our web collection on statistics for biologists contains articles on many of the points above.

Software and code

Policy information about availability of computer code

Data collection	Behavior tracking was performed with the code published in Mersch et al., 2013 (doi: 10.1126/science.1234316).

Data analysis	Behavior tracking data was analyzed with the code published in Mersch et al., 2013 (doi: 10.1126/science.1234316). Gene expression analysis was performed with MASER platform (https://cell-innovation.nig.ac.jp/maser/index_en.html) , 'edgeR' in R (ver.3.6.1), and 'clustergram' in MATLAB 2020a. Gene Ontology enrichment analyses were performed using gProfiler2 (Ensemble 103; https://biit.cs.ut.ee/gprofiler/gost). WGCNA analysis was performed with 'WGCNA' package in R. Reference genome assembly and annotation was performed with Guppy v2.1.3 (Oxford Nanopore Technologies) and wtdbg2(https://doi.org:10.1038/s41592-019-0669-3). The assembly was then polished with Nanopore long reads using Medaka v0.6 (https://github.com/nanoporetech/medaka), then with Illumina reads first using Pilon51, and then using variants called by freebayes (https://github.com/ekg/freebayes) with variant quality scores greater than 30. Candidate annotations were generated using the hint guided gene predictors Augustus (https://doi.org:10.1186/1471-2105-7-62) and SNAP(https://doi.org:10.1186/1471-2105-5-59), genome-guided RNA-seq assembly from the Trinity assembler(https://doi.org:10.1038/nprot.2013.084) and PASA(https://doi.org:10.1093/nar/gkg770) refinement tool, and liftover of NCBI RefSeq protein annotations from Camponotus floridanus using GenomeThreader(https://doi.org:10.1016/j.infsof.2005.09.005). Augustus and SNAP were run through the MAKER pipeline(https://doi.org:10.1186/1471-2105-12-491). All candidate gene models were then combined using EvidenceModeler(https://doi.org:10.1186/gb-2008-9-1-r7). In Fig. 4, Amino acid sequences of CYPS were aligned using MAFFT version 7.490 in the E-INS-i mode. Gene trees were reconstructed using RAxML version 8.1.15. Trees were visualized using Figtree version1.4.0 (http://tree.bio.ed.ac.uk/software/figtree/). qRTPCR data was analyzed with geNORM with qbase 3.2 by Biogazelle (https://www.qbaseplus.com/). Survival data was analyzed with package 'coxme' in R.

For manuscripts utilizing custom algorithms or software that are central to the research but not yet described in published literature, software must be made available to editors and reviewers. We strongly encourage code deposition in a community repository (e.g. GitHub). See the Nature Portfolio guidelines for submitting code & software for further information.

Data

Policy information about availability of data

All manuscripts must include a data availability statement. This statement should provide the following information, where applicable:
- Accession codes, unique identifiers, or web links for publicly available datasets
- A description of any restrictions on data availability
- For clinical datasets or third party data, please ensure that the statement adheres to our policy

DNA sequencing data is available at NCBI as BioProject: PRJNA901066, BioSample : SAMN31703645. RNA sequencing data is available at NCBI as BioProject: PRJDB14708, BioSample: SAMD00553588-SAMD00553647. Transcriptome data is provided at Dryad repository (https://doi.org/ 10.5061/dryad.hdr7sqvnt). Image data for Fig. 3b and 3c, and the dataset for the time points of nest entry and exit in Fig. 1a, 3c, and 4d have been deposited at Dryad repository (https://doi.org/10.5061/dryad.gb5mkkwvv).

Human research participants

Policy information about studies involving human research participants and Sex and Gender in Research.

Reporting on sex and gender	N/A
Population characteristics	N/A
Recruitment	N/A
Ethics oversight	N/A

Note that full information on the approval of the study protocol must also be provided in the manuscript.

Field-specific reporting

Please select the one below that is the best fit for your research. If you are not sure, read the appropriate sections before making your selection.

☒ Life sciences ☐ Behavioural & social sciences ☐ Ecological, evolutionary & environmental sciences

For a reference copy of the document with all sections, see nature.com/documents/nr-reporting-summary-flat.pdf

Life sciences study design

All studies must disclose on these points even when the disclosure is negative.

Sample size	Behavior analysis was performed for grouped (n=36) and isolated (n=18) ants in Figs. 1a and b. RNA seq was performed for grouped (n=42) and isolated (n=18) samples in Figs 1c-d. For qRTPCR, grouped (head&abdomen:n=44) and isolated (head:n=41,abdomen:n=40) samples were examined in body parts, and grouped (n=23) and isolated (n=22) samples were examined in tissues. For ROS quantification in Fig. 3a, grouped (n=21) and isolated(n=21) samples for DT, grouped (n=20) and isolated (n=20) samples for FB, and grouped (n=22) and isolated (n=23) samples for heads were examined. For quantification of oxidative stress marker in oenocytes in Fig. 3b, grouped (n=204) and isolated (n=214) samples for CellROX, grouped (n=206) and isolated (n=269) for 4-HNE, and grouped (n=266) and isolated (n=179) samples for SYTOX were

examined. In Fig. 3c, grouped (n=45) and isolated(n=35) samples were used to quantify the behavior and ROS level. For survival analysis in Fig. 4a, ctrl (n=101), Mel 0.1µg/ml (n=60), Mel 0.3µg/ml (n=54), Mel 1µg/ml (n=89),and Mel 10µg/ml (n=45) were tested to examine the effect of melatonin in the left graph. Ctrl (n=84), NAD 0.13µg/ml (n=63), NAD0.43µg/ml (n=63), and NAD 1.3µg/ml (n=45) were tested to examine the effect of NAD in the middle graph. Ctrl (n=60), Mel 0.3µg/ml (n=20), Mel 1µg/ml (n=59), NAD 0.13µg/ml (n=20), and NAD 0.43µg/ml (n=56) were tested to examine the effect of melatonin and NAD for grouped treatment in the right graph. For quantification of ROS with melatonin treatment in Fig.4c, ctrl (n=18) and melatonin-treated (n=19) samples were examined for heads, DTs and FB+OE. For behavior analysis with melatonin treatment in Fig. 4d, isolated-ctrl (n=38), isolated_melatonin (n=36), grouped-ctrl (n=43) and grouped-melatonin (n=40) samples were examined. No sample size calculation was performed, however we used more than 2 colonies for all experiments except the immunostaining in Fig. 3b, and used the colony background as the random factor for statistical analysis.

Data exclusions

Among the 60 samples in the RNA-seq experiment, the six grouped samples with a detection rate less than 10% were excluded from the behavioral analysis in Figs. 1a, b, f, 2a, c, e, and g.
To exclude the DEGs with outliers, we calculated the mean RPKM value from grouped (42 samples) and isolated (18 samples) treatments, and the ratio of RPKM between isolated and grouped treatment (referred as ratio with all samples). We next calculated the mean RPKM value in isolated and grouped treatments excluding the minimum and maximum values of RPKM in each social treatment, and then calculated the ratio of RPKM without the minimum and maximum values between isolated and grouped treatment (referred as ratio without max/min). We then excluded the genes with an absolute log2 ratio (ratio with all samples/ratio without max/min) >1 as outliers. For the volcano plot, genes with mean RPKM > 1.5 and median RPKM > 0 were plotted.

Replication

RNAseq was the results of one experiments. qRTPCR was the summary of 3 (for parts samples) or 4 (for tissue samples) repeated experiments. ROS quantification in Fig. 3a was the summary of independent 4 experiments. Quantification of oxidative stress marker in Fig. 3b is the summary of independent 2 experiments for each parameter. ROS intensity and behavioral parameters in Fig. 3c was the summary of independent 4 experiments. Survival test in Fig. 4a is the summary of independent 7, 6 or 3 experiments in isolated-melatonin, isolated-NAD, or grouped conditions. ROS quantification in Fig 4c is the summary of 2 independent experiments. Behavior assay with melatonin in Fig. 4c is the summary of 9 independent experiments. In all experiments, all attempts at replication were successful.

Randomization

Samples were randomly allocated for each treatment (grouped or isolated). For each experiment (except data in Fig. 3b), the experimental colonies were created using 2-4 independent colonies of origin (Fig. 1 RNA-seq: 3 colonies; Fig. 2 qRTPCR for parts: 3 colonies; Fig. 2 qRTPCR for tissues: 2 colonies; Fig. 3a ROS quantification: 3 colonies; Fig. 3c ROS and behavior quantification: 3 colonies;Fig. 4a Survival with Mel: 4 colonies; Fig. 4a Survival with NAD: 3 colonies; Fig. 4a Survival in grouped: 4 colonies; Fig. 4c ROS quantification: 3 colonies; Fig. 4d Behavior: 3 colonies). Colony-of-origin effects were accounted for analytically (see below). All individuals for the immunohistochemistry in Fig. 3b originated from the same colony.

Blinding

Behavior data collection and analyses were performed with automated procedure for behavior tracking (Fig. 1a-c, 2a, 2c, 2e, 2g, and 4d) and gene expression analysis (Fig. 1d-h, 2a, 2c, 2e, and 2g). Therefore it were not subject to human biases. For qRTPCR (Fig. 2b, 2d, 2f, and 2h) and ROS quantification experiments (Fig. 3a and 4c), the blinding was impossible for the sample collection, however the data was normalized against multiple reference genes with automated program (qbase software) or the amount of protein, and therefore was not subject to human biases. Blind test was performed for the quantification of signal intensity in Fig. 3b and 3c. The blinding was not possible for survival tests (Fig. 4a), however we checked the status of each ant every day and the time of death was defined as when the body lay motionless, which was not subject to human biases.

Reporting for specific materials, systems and methods

We require information from authors about some types of materials, experimental systems and methods used in many studies. Here, indicate whether each material, system or method listed is relevant to your study. If you are not sure if a list item applies to your research, read the appropriate section before selecting a response.

Materials & experimental systems

n/a	Involved in the study	
☐	☒	Antibodies
☒	☐	Eukaryotic cell lines
☒	☐	Palaeontology and archaeology
☐	☒	Animals and other organisms
☒	☐	Clinical data
☒	☐	Dual use research of concern

Methods

n/a	Involved in the study	
☒	☐	ChIP-seq
☒	☐	Flow cytometry
☒	☐	MRI-based neuroimaging

Antibodies

Antibodies used

anti 4-Hydroxynonenal antibody (ab46545, abcam)
Alexa 488-conjugated anti-rabbit secondary antibody (A-21206, Invitrogen)

Validation

https://www.abcam.co.jp/4-hydroxynonenal-antibody-ab46545.html
https://www.thermofisher.com/antibody/product/Donkey-anti-Rabbit-IgG-H-L-Highly-Cross-Adsorbed-Secondary-Antibody-Polyclonal/A-21206

Animals and other research organisms

Policy information about studies involving animals; ARRIVE guidelines recommended for reporting animal research, and Sex and Gender in Research

Laboratory animals	Camponotus fellah colonies were initiated from queens collected after a mating flight in March 2007 or 2010 in Tel Aviv, Israel, then reared in the laboratory condition more than 10 years. In this study, we used worker ants born in the laboratory from the colony raised above. The ants were reared in an incubator (NIPPON MEDICAL & CHEMICAL INSTRUMENTS CO., LTD) under controlled conditions (12:12 LD, 30°C, 60% RH) with food (made from honey, eggs, and vitamin tablets), water, and a light-shielded nest box. The workers for experiments were killed and sampled, however the original colony is kept after the experiments.
Wild animals	None
Reporting on sex	All ant workers used were female.
Field-collected samples	None
Ethics oversight	No ethical approval or guidance was required for the use of insects in research.

Note that full information on the approval of the study protocol must also be provided in the manuscript.
